# From Tank to Treatment: Modeling Melanoma in Zebrafish

**DOI:** 10.3390/cells9051289

**Published:** 2020-05-22

**Authors:** William Tyler Frantz, Craig J Ceol

**Affiliations:** 1 Program in Molecular Medicine, University of Massachusetts Medical School, Worcester, MA 01605, USA; William.Frantz@umassmed.edu; 2 Department of Molecular, Cell, and Cancer Biology, University of Massachusetts Medical School, Worcester, MA 01605, USA

**Keywords:** Melanoma, zebrafish, melanocytes, modeling, genetics, microenvironment, xenografts

## Abstract

Melanoma is the deadliest form of skin cancer and one of few cancers with a growing incidence. A thorough understanding of its pathogenesis is fundamental to developing new strategies to combat mortality and morbidity. Zebrafish—due in large part to their tractable genetics, conserved pathways, and optical properties—have emerged as an excellent system to model melanoma. Zebrafish have been used to study melanoma from a single tumor initiating cell, through metastasis, remission, and finally into relapse. In this review, we examine seminal zebrafish studies that have advanced our understanding of melanoma.

## 1. Melanoma in Humans

Melanoma is a lethal malignancy of the melanocytes, cells that derive from the neural crest, produce melanin, and are most commonly found in the skin. Melanoma accounts for only 1% of skin cancer cases, but is responsible for most skin cancer deaths [1,2]. The World Health Organization estimates that in 2018, 287,723 new cases of cutaneous melanoma were diagnosed and 60,712 patients succumbed to their disease worldwide [2]. It is also estimated that there will be 4% more new diagnoses of cutaneous melanoma in 2020 than in 2019, continuing the 47% rise seen over the last decade [1]. The vast majority of melanoma is caught early before it invades surrounding structures. These stage 1 and 2 melanomas have a 98% five-year survival rate. However, the survival rates drop precipitously for stage 3 and 4 melanomas. Stage 4 melanomas, which are characterized by distant metastatic spread, have an aggressive course of disease and poor prognosis [1].

In 2011, vemurafenib, a small molecule inhibitor that targets the most common mutation in melanoma, B-Raf proto-oncogene, serine/threonine kinase (BRAF^V600E^), became the first drug since interleukin (IL)-2 therapy to show improvement in treating melanoma [3,4,5]. Unfortunately, the majority of patients on BRAF inhibitor monotherapy relapsed and died with little improvement in survival [5]. More recently, combination therapy trials revealed that patients positive for oncogenic *BRAF* mutations treated with the BRAF inhibitor dabrafenib plus the dual specificity mitogen-activated protein kinase kinase 1 (MAP2K1/MEK) inhibitor trametinib had improved survival [6]. Immunotherapies have been pioneered in melanoma and have produced more durable remissions. The Checkmate 067 trial investigated the impact of treatment of metastatic melanoma with the cytotoxic T-lymphocyte-associated protein 4 (CTLA-4) inhibitor ipilimumab alone, the programmed death-ligand 1 (PD-L1) inhibitor nivolumab alone, and combination therapy [7]. The trial demonstrated superior survival outcomes for combination immunotherapy vs. single immunotherapy. At the five-year mark, 52% of patients on combination ipilimumab/nivolumab were still alive (44% for nivolumab alone and 26% for ipilimumab alone) [7]. These clinical trials demonstrated substantial improvements in patient outcomes compared to a decade ago and are currently being extended to the adjuvant setting [8]. However, despite these improvements, nearly half of patients still succumbed to disease in five years, and adverse side effects were a substantial problem with combined immunotherapy. While the success of BRAF inhibitors and immunotherapy in improving outcomes is encouraging, the high rates of resistance and relapse underscore the need for further research.

Identifying the genetic, molecular, and cellular pathobiology of melanoma is fundamental to improving our diagnostic tools and developing novel therapeutics. Over the last two decades zebrafish have become an established model and an excellent platform for such studies. In this review, we will detail seminal zebrafish studies that have advanced our understanding not only of melanomagenesis and disease progression, but also provided the basis for therapeutic development. 

## 2. Melanocytes in Zebrafish

Zebrafish were first introduced as a model organism nearly 40 years ago, primarily for their utility in developmental biology research [9]. Over the past decade, zebrafish have become an important model organism for studying disease and development. Zebrafish have been used to model disparate disease processes from cancer to infection [10,11]. The range of organ systems in the zebrafish allows for modeling diverse cancers, ranging from hematopoietic malignancies such as leukemia to solid tumors such as melanoma [12,13].

**Figure 1:**
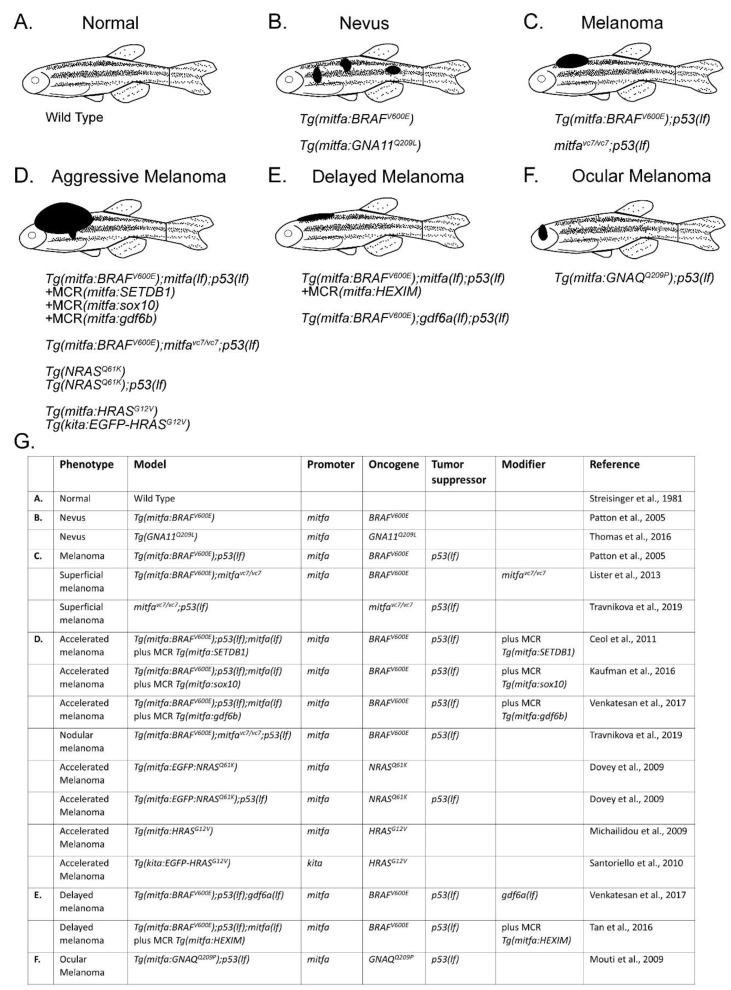
Genetic models of melanoma in zebrafish. (**A**) Normal melanocyte pattern in wild-type zebrafish. (**B**) Nevus formation with the introduction of human B-Raf proto-oncogene, serine/threonine kinase (*BRAF^V600E^*) or G protein subunit alpha 11 (*GNA11^Q209L^*) under the zebrafish microphthalmia-associated transcription factor alpha (*mitfa*) promoter [13,44]. (**C**) Melanoma formation with the introduction of human *BRAF^V600E^* in a *p53* loss-of-function *(lf)* or *mitfa(vc7)* background [13,45]. (**D**) Melanoma modifiers introduced into the *Tg(mitfa:BRAF^V600E^); p53(lf)* model using MiniCoopR (MCR) resulted in aggressive melanomas, comparable to other models with alternative tumor drivers such as NRAS proto-oncogene, GTPase (*NRAS^Q61K^*) or HRas proto-oncogene, GTPase (*HRAS^G12V^*) [21,27,36,45,46,47,48]. (**E**) Expression of HEXIM P-TEFb complex subunit 1 (*HEXIM1*) using MiniCoopR, or loss of growth differentiation factor 6 alpha (*gdf6a*) in a *Tg(mitfa:BRAF^V600E^);p53(lf)* background led to delayed melanoma onset [36,49]. (**F**) Ocular melanoma resulted from introduction of human G protein subunit alpha q (*GNAQ^Q209P^*) in a *p53* loss-of-function background [32]. (**G**) Table of zebrafish melanoma model components from **A**–**F**.

Zebrafish melanocytes derive from the neural crest and differentiate into large, dendritic, melanized cells. There are dermal melanocytes arranged in a series of lateral stripes, giving rise to their characteristic namesake patterning (Figure 1A). Zebrafish also have scale-associated melanocytes which develop from the neural crest and are prone to transformation in adult zebrafish melanoma models. Recently, adult melanocyte stem cells (MSCs) were also identified in zebrafish [14]. This pool of stem cells is admixed with mature melanocytes inside the melanocyte stripe. These stem cells respond to injury by differentiating into mature melanocytes to reconstitute the skin’s pigment pattern or dividing symmetrically to replenish the melanocyte stem cell pool. Whereas zebrafish melanocytes share many features with human melanocytes, there are differences that may limit the use of zebrafish in studying melanocyte and melanoma biology. Unlike their mammalian counterparts which impart pigment-containing melanosomes to the surrounding keratinocytes, zebrafish melanocytes retain their melanosomes. Additionally, the skin architecture and niches in which melanocytes and MSCs reside is considerably different between species. Mammalian MSCs reside primarily in the bulge region of the hair follicle, where they replenish melanocytes in the hair follicle bulb and the epidermis. By contrast, stripe melanocytes in zebrafish are interspersed throughout the hypodermis without apparent association to any anatomical niche [15].

In addition to their genetic tractability, zebrafish possess desirable optical properties. Zebrafish embryos and larvae are transparent, allowing time-lapse imaging of developmental processes ex vivo. Wild-type adult zebrafish are more opaque, although superficial cells such as melanocytes are readily visualized. This superficial location, along with their melanin retention, allows precise assaying of melanocyte cells at single-cell resolution in live animals.

The construction of the first zebrafish reference genome revealed that zebrafish contain 26,000 protein coding genes and 71.4% of human genes have an obvious zebrafish ortholog [16]. This percentage is higher for human disease-related genes, with 82% of such genes having a zebrafish ortholog [16]. The pathways involved in zebrafish melanocyte biology are highly conserved. Zebrafish melanocyte development is dependent on an *MITF* ortholog as a master regulator, *KIT* and other mitogen-activated protein kinase (MAPK) signaling genes as important executors of cell fate, and a host of melanin biosynthesis genes (including *TYR*, *TYRP1*, and *PMEL*) for differentiation. Loss of the zebrafish orthologs of these genes results in phenotypes identical or similar to those found in mammals. For instance, zebrafish with a mutation in tyrosinase fail to produce melanin, mirroring the mechanism for human albinism [17,18]. Conserved molecular and genetic pathways make zebrafish a suitable model to study melanocyte biology and pathologies, including melanoma.

## 3. Modeling Melanoma Disease Drivers in Zebrafish

Activating mutations in *BRAF*, most commonly *BRAF^V600E^*, occur in nearly 50% of human melanoma and lead to overactivation of the MAPK pathway [19]. However, these mutations alone are not sufficient to give rise to malignancy, as human nevi frequently express *BRAF^V600E^* [20]. To test whether *BRAF^V600E^* could formally promote melanoma, Patton and colleagues expressed human *BRAF^V600E^* under the melanocyte-specific *mitfa* promoter and found that zebrafish only developed nevi [13]. However, injection into *p53* loss-of-function mutants, *p53(lf),* gave rise to tumors that histopathologically mirrored human melanomas [13]. Similarly, Dovey and colleagues successfully modeled *NRAS* melanomas by introducing oncogenic *NRAS^Q61K^* into *p53(lf)* animals [21]. While *p53* itself is only mutated in 19% of human melanomas, most human melanomas lose *p53* pathway function due to other mutational events, including frequent loss of the *CDKN2A* locus, which encodes the *ARF* tumor suppressor, a regulator of *p53* [22,23,24,25,26]. Additional models involving less frequently mutated driver genes have been designed, including oncogenic *HRAS*-dependent models [27,28,29]. Rarer melanoma subtypes have also been modeled in zebrafish. Uveal melanomas are biologically distinct from their cutaneous counterparts and often contain driver mutations in the G protein subunits *GNAQ* or *GNA11* [30,31]. In 2016, Mouti and colleagues created the first zebrafish model of uveal melanoma by driving the *GNAQ^Q209P^* oncogene under the *mitfa* promoter in a *p53(lf)* background [32]. They found that 33% of these *Tg(mitfa:GNAQ^Q209P^); p53(lf)* animals go on to develop uveal melanoma [32]. Based on similar genetics and histopathologic features, these zebrafish melanomas are faithful representatives of human melanoma (Figure 1B–G).

Genomic features of zebrafish melanoma and their similarities to human melanoma have been described. Zebrafish *BRAF* and *NRAS*-dependent melanomas do not display the same frequencies of mutations as seen in sun-exposed human melanoma [33,34]. This is not unexpected, as human melanomas retain a history of UV-induced mutations present in the cell of origin prior to transformation, and such UV exposure is not present in zebrafish housing tanks. By contrast, the high degree of copy number variation in human melanomas is also observed in zebrafish tumors [34,35,36]. Because of their preponderance, it is likely that these copy number variations are important in the initiation and further progression of zebrafish melanomas. In support of this possibility, many genes subject to copy number variation in zebrafish melanoma are similarly varied in their copy number in human melanoma [36]. A high degree of copy number alterations may be a common feature of zebrafish tumor models, as malignant peripheral nerve sheath tumors caused by loss of *p53* also have frequent copy number alterations [37].

The creation of these melanoma models has underscored the conserved nature of the pathway activity changes between zebrafish and human melanomas. Consequently, zebrafish melanomas, based on insights gained from patient sequencing results, serve as the platform to investigate additional genetic and environmental melanoma modifiers of initiation, progression, metastasis and treatment.

## 4. Using Zebrafish to Discover Genetic Modifiers of Melanoma

These zebrafish melanoma models present the opportunity to investigate melanoma in a controlled environment. In all these models, there is a cancerized field of cells with the same genetic alterations. However, most cells will fail to give rise to tumors. Furthermore, even the cells that eventually become tumors take many months to progress. This indicates the need for additional genetic or epigenetic alterations for tumor formation and presents the opportunity for zebrafish tumorigenic models to serve as poised backgrounds for the discovery of new melanoma modifier genes [38,39]. Some of these modifiers are likely present in the many available genome sequences from patient melanomas. However, melanomas have among the highest mutation burdens of any cancer [19,40,41,42,43], and most of the detected mutations and copy number variations in human melanomas are likely to be passenger alterations. Using zebrafish as a screening platform, with its high throughput and tumorigenesis as a readout, the few driver genes can potentially be discerned from the many passenger genes present in a patient’s melanoma sequence.

The first use of zebrafish to screen for novel genetic modifiers focused on recurrently amplified areas of the human melanoma genome [46]. In this study, a ‘MiniCoopR’ screening strategy was developed that utilized a strain in which a *mitfa* loss-of-function mutation was introduced into a tumor-prone *Tg(mitfa:BRAF^V600E^); p53(lf)* background. The *mitfa* mutation abrogated melanocyte development and melanoma formation. In *Tg(mitfa:BRAF^V600E^); mitfa(lf); p53(lf)* animals, a transgene containing a wild-type *mitfa* gene was introduced from the MiniCoopR vector, thus rescuing melanocytes and melanomas. This same vector also introduced a companion gene whose effects on tumor initiation and progression could be measured in rescued animals. Among the companion genes screened were several from recurrently amplified regions of the melanoma genome. One gene from a recurrently amplified interval of chromosome 1q [50,51], SET domain bifurcated histone lysine methyltransferase 1 (*SETDB1)*, significantly accelerated melanoma onset. Mechanistic studies revealed that higher expression of *SETDB1* allowed melanocytes to escape senescence in response to oncogenic insult. Importantly, immunohistochemistry of human tissues showed that SETDB1 protein was highly expressed in melanomas as compared to benign nevi or normal melanocytes [46]. This discovery established a powerful method for using zebrafish to identify potential oncogenes from genomic and transcriptional changes present in human tumors.

Variations of the MiniCoopR strategy, described above, have been developed to screen for melanoma modifiers. Recently, Ablain and colleagues utilized the MiniCoopR platform to identify a tumor suppressor of mucosal melanoma. After sequencing human mucosal melanomas, they learned that sprout related EVH1 domain containing 1 (SPRED1), a negative regulator of the MAPK pathway, is inactivated in 37% of these tumors [52]. A modified version of the MiniCoopR approach was developed to test the effect of *SPRED1* loss on melanoma initiation [53]. This new system, termed Mazerati, combined *mitfa*-dependent melanocyte rescue with Crispr/Cas9-mediated genome editing [53]. Zebrafish *spred1* loss was found to accelerate melanoma onset in a *KIT*, but not *BRAF* or *NRAS*, oncogene background. Since *KIT* mutations predominate over *BRAF* or *NRAS* mutations in mucosal melanomas, this result suggests a special cooperativity between *KIT* gain of function and *SPRED1* loss.

Melanoma genes have also been identified based on comparative genomic approaches. Venkatesan and colleagues compared genes subject to copy number amplification in human melanomas to those subject to copy number amplification in zebrafish melanomas [36]. This comparison revealed that many syntenic genomic intervals, and the genes within these intervals, are amplified in both species, suggesting an underlying mechanistic conservation of melanomagenesis. In MiniCoopR-based screening of genes amplified in both species, the bone morphogenetic protein (BMP) ligand *GDF6* (*gdf6a* and *gdf6b* in zebrafish) was identified as a new melanoma oncogene. *GDF6*-dependent BMP signaling was found to be critical for maintaining neural crest identity, and withdrawal of BMP signaling led to differentiation and death of melanoma cells. The findings that *GDF6*-dependent BMP signaling is observed in ~80% of patient tumors and its expression is correlated with patient survival imply that this gene and signaling pathway are potential targets for anti-melanoma therapy.

The strategy of using zebrafish to functionally analyze genes highlighted by genomic and transcriptomic studies of human tumors has also revealed novel insights into how melanomas cope with nucleotide stress. Tan and colleagues utilized Oncomine expression data to evaluate the role of candidate transcription elongation regulators in melanoma and found that HEXIM1 P-TEFb complex subunit 1 (*HEXIM1)* expression was significantly reduced in melanomas compared to nevi [49,54]. Consistent with the possibility that *HEXIM1* acts as a tumor suppressor, elevated expression of this gene using the MiniCoopR system delayed and Crispr/Cas9-mediated knockout accelerated melanoma onset. Mechanistic studies pointed to a role in transcriptional elongation; HEXIM1 normally acts through positive transcription elongation factor (p-TEFb) to inhibit transcription of tumor-promoting genes and stabilize transcription of other tumor suppressor genes [55]. *HEXIM1* expression itself is regulated by nucleotide stress via the specificity protein 1 (SP1) transcription factor, thus linking the state of nucleotide stores to tumorigenic potential.

The zebrafish model has uncovered the role of chromatin modifiers in melanoma. In 2017, Scahill and colleagues found that loss of *kdm2aa*, an ortholog of the histone demethylase *KDM2A*, led to the formation of spontaneous melanomas at high frequency [56]. These tumors arose independently of *BRAF^V600E^* and other common driver mutations as well as common tumor suppressor losses. Gene expression profiling showed a concerted response of genes related to translation, DNA replication, and chromatin regulation upon knockdown of *kdm2aa*, suggesting a role for aberrant chromatin methylation in melanomagenesis.

Zebrafish models have also been used to investigate genes and transcriptomic programs important in melanoma progression. Salhi and colleagues compared expression and phosphorylation patterns of tumorigenic pathways in human melanoma lines derived from radial (RGP) or vertical (VGP) growth phase melanomas and discovered that ribosomal protein S6 kinase 1 (RSK1) is active in VGP but not in RGP melanomas [57]. Subsequent cell culture assays revealed that inhibition of RSK1 led to downregulation of a transcriptional program that supports cell motility. Constitutive activation of RSK1 in zebrafish accelerated melanoma onset and promoted invasion, whereas inactivation of RSK1 delayed tumor initiation. This work provides further insight into the role of RSK1, which had been previously identified as a downstream target of the MAPK pathway, in melanoma progression [58].

Further transcriptional analysis of aggressive RSK1-activated melanomas in zebrafish revealed upregulation of genes involved in oxidative phosphorylation (OXPHOS) [59]. To investigate this association between OXPHOS and melanoma, Salhi and colleagues stained human primary melanoma samples for peroxisome proliferator activated receptor gamma coactivator 1 alpha (PGC1α), the proposed master regulator of tumor OXPHOS [60,61]. They found a positive association between PGC1α, primary melanoma thickness, and proliferative marker Ki-67, suggesting a role for PGC1α in melanoma progression. Follow up in vitro knockdowns of PGC1α resulted in downregulation of transcriptional signatures associated with melanoma progression. These results suggest a role for PGC1α in melanoma progression via mediation of oxidative phosphorylation [59].

More recently, Henderson and colleagues compared the transcriptomes of advanced VGP melanomas in zebrafish to early RGP melanomas and found that lipid metabolism was dysregulated in more advanced tumors [62]. Elevated expression of one dysregulated gene, the lipoprotein lipase *LPL*, accelerated melanoma onset in zebrafish. *LPL* has previously been implicated in tumor progression [63], and results in zebrafish support the notion that lipid metabolism can regulate even the earliest stages of tumor progression.

Taken together, the studies highlighted above underline the utility of zebrafish to screen and discover novel melanoma genes, increasing our understanding of genetic factors that drive patient outcomes.

## 5. Intersection of Melanocyte Development and Melanoma in Zebrafish

The microphthalmia-associated transcription factor (MITF) and KIT proto-oncogene, receptor tyrosine kinase (c-KIT) were two of the first genes discovered to play a role in melanocyte biology [64,65,66]. In addition to their conserved roles in development, both genes are implicated in melanoma pathogenesis [67,68,69]. These genes and the networks in which they function are well conserved, and studies in zebrafish have helped to elucidate new roles in both melanocyte development and melanoma.

MITF is the master regulator of the melanocyte lineage [70]. Evidence for this role includes genetic studies in mice and zebrafish as well as the pigmentary defects seen in *MITF*-mutant patients, who suffer from Waardenburg syndrome (type 2a). Waardenburg syndrome patients frequently display pigment defects such as white forelock, skin hypopigmentation, and premature hair graying [71,72]. More severe mutations have been isolated in mice and zebrafish, and *MITF*-null mutations in both species cause a complete loss of melanocytes [73,74]. In melanomas, early genomic studies found that *MITF* was amplified in metastatic disease and its increased expression correlated with decreased patient survival, suggesting that *MITF* is an oncogene [67]. However, several studies have shown that decreased *MITF* can also be pro-tumorigenic, indicating divergent roles for this gene in melanoma [75,76]. To address how different levels of *MITF* activity could affect melanoma initiation and maintenance, Travnickova and colleagues utilized a unique, temperature-sensitive allele of zebrafish *mitfa*. This allele, *vc7*, is hypomorphic at the permissive temperature but completely inactive at the restrictive temperature [45,77]. They found that *mitfa(vc7); p53(lf)* animals develop superficial melanomas enriched for stem and invasive gene signatures, similar to what is observed in human MITF-low melanomas [48]. Further reduction of *mitfa* activity by upshifting to the restrictive temperature led to near-total tumor regression. A small population of tumor cells, akin to minimal residual disease (MRD), remained at the tumor site, and this population seeded tumor relapse when *mitfa* activity was restored. This study indicates that *MITF* activity is important in bulk tumor cells, but MRD is characterized by *MITF* inactivity, suggesting how differing levels of *MITF* can support tumor maintenance and relapse.

The type 3 receptor tyrosine kinase KIT is required for normal melanocyte development in mammals and zebrafish. In humans, heterozygous loss of the *KIT* gene results in piebaldism, a pigmentation disorder characterized by a white forelock [78]. Mice deficient in *Kit* and its cognate ligand, stem cell factor (SCF), develop a similar pigmentation phenotype of white spotting [66,79]. Zebrafish orthologs of *KIT* and its ligand (*kita* and *kitlga*, respectively) are deficient in melanocytes due to their inappropriate death in embryos and failure to develop in adults [80,81]. *KIT* gain-of-function mutations have been shown to drive the formation of rare acral and mucosal melanoma subtypes [68,69]. By contrast, more common cutaneous melanomas typically show loss of *KIT* expression [82,83]. The effect of *KIT* loss was investigated in zebrafish by introducing a *kita*-null mutation into the *Tg(mitfa:BRAFV600E); p53(lf)* melanoma-prone strain [84]. Loss of *kita* caused accelerated melanoma onset, with increased BRAF^V600E^-driven MAPK pathway signaling evident in tumors. Mechanistic studies revealed that KIT engages wild-type BRAF, which competes with BRAF^V600E^, thereby attenuating signaling flux through the MAPK pathway [84].

Studies with MITF and KIT take advantage of the conserved genetic, molecular, and cellular behaviors of mammalian and zebrafish melanocytes, making zebrafish an excellent platform for discovering new insights into melanocytes and their pathologies.

## 6. Zebrafish Neural Crest Reactivation in Melanoma Initiation and Progression

The neural crest is a highly migratory, multipotent embryonic cell population that gives rise to diverse cell types including cutaneous melanocytes. Early transcriptional studies discovered that aggressive melanoma cells have gene signatures, like those of neural crest cells, associated with cellular plasticity, dedifferentiation and migration [85,86,87,88]. In vivo studies revealed that melanoma cells transplanted into zebrafish embryos maintain a plastic, dedifferentiated, pro-migratory state [89]. Similarly, melanoma cells transplanted into chick embryos invaded along stereotypical neural crest migratory pathways, suggesting these cells respond to neural crest microenvironmental signals [90].

Zebrafish models have been vital in understanding the importance and timing of this neural crest gene program. In 2011, White and colleagues discovered that zebrafish melanomas reactivated a developmental transcriptional signature highlighted by neural crest genes [91]. To determine if these transcriptional changes were important for melanoma progression, a chemical genetic screen was performed for small molecules capable of suppressing neural crest development in zebrafish embryos. Dihydroorotate dehydrogenase inhibitors, including the FDA-approved drug leflunomide, inhibited neural crest development via the reduction of pyrimidine reserves, resulting in pausing of transcriptional elongation in genes required for neural crest development [91]. A mechanistic follow up study utilized a chemical screen for the rescue of neural crest development following leflunomide treatment and identified the RNA helicase protein DExD-box helicase 21 (DDX21) as a sensor and mediator of transcription during nucleotide stress [92]. Crucially, treatment with leflunomide caused a decrease in melanoma cell growth in vitro, in addition to inhibiting the growth of autochthonous zebrafish melanomas and xenografted melanomas in mice [91]. Clinical trials are currently testing the effects of leflunomide in combination with other melanoma treatments such as MEK inhibitors [93].

In 2016, Kaufman and colleagues used a reporter for a key member of this neural crest signature, the gene *crestin,* to show that it was expressed in early-stage melanomas [47]. Indeed, *crestin* expression was observed in single cells of origin that would progress to form tumors. The *crestin* reporter thus serves as a useful tool to track, in real time, the earliest stages of tumor initiation [47,94]. While there is currently no known mammalian equivalent of *crestin*, this landmark discovery has given rise to new research into the genetic and epigenetic mechanisms governing a neural crest state reactivation.

More recently, the *crestin* reporter has been utilized in a chemical genetic screen to identify modulators of neural crest identity [95]. In this screen, caffeic acid phenethyl ester (CAPE) was found as an inhibitor of zebrafish neural crest development. Mechanistically, CAPE interrupted sox10-mediated neural crest formation via inhibition of the fibroblast growth factor-stimulated phosphoinositide 3-kinase/Ak strain transforming (PI3K/AKT) signaling axis [95]. This identification of PI3K/AKT as a novel intracellular pathway regulating neural crest differentiation provides further targets for manipulating neural crest identity in melanoma.

These zebrafish melanoma studies have revealed that a neural crest gene signature, marked by *crestin*, is present in the earliest melanoma lesions and remains present through disease progression. Importantly, utilizing high throughput drug screening of embryonic phenotypes, such as neural crest development, has identified pathway inhibitors with potential anti-tumor properties [91,95]. This new understanding of the importance and timing of melanoma’s neural crest identity coupled with high throughput screening can enable accelerated discovery of novel melanoma therapeutics.

## 7. Utilizing Zebrafish to Understand the Melanoma Microenvironment

Melanoma lethality is due in part to its proclivity to progress and metastasize into difficult-to-treat areas such as the lungs, brain and bone. Metastatic melanoma cells must migrate through complex tissues, seed, and then survive in distant tissues. Understanding how other cells influence melanoma initiation, progression, and metastasis is critically important for improving outcomes for melanoma patients. The desirable optical properties of zebrafish coupled with plentiful tissue-specific reporters allow for in vivo visualization of interactions between melanoma cells and their surrounding tissues, thus enabling studies of the melanoma microenvironment.

### 7.1. Using Zebrafish to Study Phenotype Switching in Melanoma

**Figure 2:**
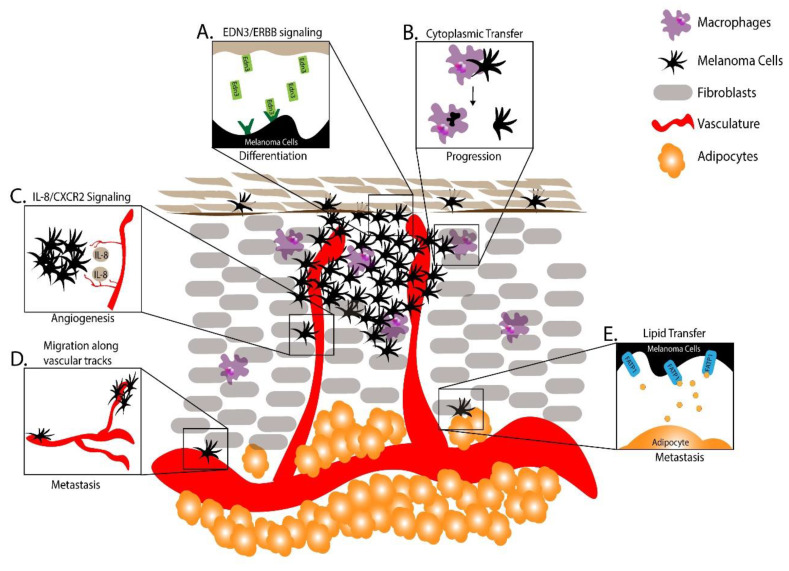
Microenvironmental factors governing melanoma progression from zebrafish. (**A**) Endothelin 3/erb-b2 receptor tyrosine kinase (EDN3/ERBB) signaling promoted differentiation and proliferation upon metastasis [97]. (**B**) Melanoma cell cytoplasmic transfer to macrophages promoted melanoma progression [99]. (**C**) Interleukin 8/C-X-C motif chemokine receptor 2 (IL-8/CXCR2) signaling encouraged melanoma progression [103,104]. (**D**) Melanoma cells cupped vasculature to migrate and metastasize [105]. (**E**) Adipocytes contributed fatty acids to melanoma cells during melanoma progression [106].

During the metastatic process melanoma cells are thought to undergo phenotype switching, in which a dedifferentiated, less proliferative and more motile phenotype is adopted to initiate metastasis, and a more differentiated and proliferative phenotype is adopted to facilitate growth of macrometastases upon seeding into distant tissues. To understand phenotype switching, Kim and colleagues injected a zebrafish melanoma cell line, ZMEL1-GFP, into pigmentless “*casper*” zebrafish to model melanoma metastasis [96,97]. Using this model, individual metastatic cells were tracked and metastatic spread and growth quantified longitudinally [98]. Upon injection, ZMEL1-GFP cells, which are unpigmented and mesenchymal in culture, became pigmented at the engraftment site. Similarly, secondary metastases appeared unpigmented at first but soon became pigmented, suggesting that signals at the site of metastasis were regulating a phenotype switch. Gene expression profiling combined with in vitro screening revealed that the vasoactive endothelins (EDN)-1 and EDN-3 induced differentiation and proliferation. Consistent with a role in promoting differentiation and proliferation upon metastatic spread, ZMEL1-GFP secondary metastases in zebrafish mutant for *edn3* and its biosynthetic enzyme *ece2b* were smaller and less pigmented. This work uncovered a novel signaling system governing phenotype switching in target tissues during melanoma metastasis (Figure 2A) [97].

### 7.2. Using Zebrafish to Study Innate Immune Cells in Melanoma

Tissue-resident macrophages serve a variety of functions such as tissue remodeling, phagocytosis, and antigen presentation. Macrophages are also present in tumors where their functions are less understood. To investigate the role of macrophages in melanoma, Roh-Johnson and colleagues transplanted melanoma cells into larval zebrafish and monitored tumor–immune cell interactions [99]. Surprisingly, microscopy revealed that GFP-labeled melanoma cells transferred a portion of their cytoplasm to the macrophages, and this cytoplasmic transfer correlated with melanoma cell dissemination. Follow-up studies revealed that blocking macrophage recruitment to transplanted melanoma cells decreased tumor cell dissemination, indicating that this melanoma–macrophage cytoplasmic transfer is functionally important to metastatic progression (Figure 2B).

Macrophages also play key roles in inflammation and cancer [100]. Recently, Gόmez-Abenza and colleagues discovered that serine peptidase inhibitor, Kunitz type 1 (SPINT1) regulated melanoma aggression and crosstalk in the tumor microenvironment [101]. They noted that a subset of melanoma patients had high levels of SPINT1 mRNA, and this upregulation correlated with poor prognosis and greater tumor-associated macrophage infiltration. Furthermore, more aggressive melanomas were observed in a SPINT1-deficient background in zebrafish, suggesting that SPINT1 deficiency accelerates melanoma formation via altered immune cell recruitment and activity [101].

### 7.3. Using Zebrafish to Study Vascular Cells in Melanoma

Angiogenesis is a critical step in the progression of microtumors. In zebrafish, the growth of new tumor vasculature has been studied by injecting melanoma cells into the perivitelline space of embryos and observing the recruitment and growth of fluorescently-labeled vasculature [102]. More recent mechanistic studies have investigated signals that regulate angiogenic growth [103,104]. In addition to vascular endothelial growth factor (VEGF)-mediated signaling, it was shown that vasculature-derived IL-8 could signal through the C-X-C motif chemokine receptor 2 (CXCR2) receptor in melanoma cells to promote angiogenesis. Taken together, these studies highlight the mechanisms of cell–cell signaling between melanomas and vasculature to promote melanomagenesis (Figure 2C).

To model the migratory potential of melanoma cells, Fornabaio and colleagues injected GFP-labeled cutaneous melanoma cells and non-malignant melanocytes into larval zebrafish yolk sacs and monitored their survival and migration [105]. Strikingly, the melanoma cells migrated outside of the yolk sac while the non-malignant cells either died or remained in the yolk sac. Time lapse imaging coupled with 3D reconstruction revealed these migrating melanoma cells changed their morphology to extend pseudopods and cup the external surface of blood vessels [105]. Uveal melanoma cell lines revealed a similar angiotropism, demonstrating that cutaneous and uveal melanomas can utilize vasculature as tracts for migration and metastasis even without intravasation (Figure 2D).

### 7.4. Using Zebrafish to Study Adipocytes in Melanoma

As melanoma advances, it typically grows through adipocyte-rich subcutaneous tissue, introducing a new microenvironment and different cell–cell interactions. Recently, Zhang and colleagues used a combination of mouse and zebrafish models to investigate how adipocytes promote melanoma progression. They found that adipocytes in the melanoma microenvironment transferred fatty acids to melanocytes via fatty acid transport protein 1 (FATP1) transporters expressed on the surface of melanoma cells [106]. Furthermore, elevated expression of FATP1 expression in melanocytes accelerated BRAF^V600E^-driven melanoma development in both zebrafish and murine models. This work highlighted the utility of zebrafish and murine models to uncover complex melanoma microenvironmental interactions promoting the progression of melanoma.

The melanoma microenvironment is complex, dynamic, and diverse. Melanoma utilizes or interrupts signals from surrounding fibroblasts, vasculature, immune cells, adipocytes and other cell types in order to survive, proliferate and invade. With several autochthonous models in which tumor-microenvironment interactions can be measured and manipulated, studies in zebrafish are likely to further expand our understanding of how the microenvironment encourages the initiation, progression and metastasis of melanoma.

## 8. Future Directions

While treatments for late-stage melanoma have greatly improved in the last decade, significant challenges remain in elucidating disease and drug resistance mechanisms and translating these findings to a clinical environment to benefit patients who respond poorly to current therapies. This group of poor responders is evidenced by the nearly 50% of metastatic melanoma patients who still die within five years of diagnosis [6,7]. Zebrafish models have provided, and can continue to provide, unique insights into pathogenesis and treatment.

### 8.1. Understanding the Micro- and Macro-Environmental Factors that Encourage Disease Initiation, Progression, Metastasis and Relapse

Deeper knowledge of how the melanoma microenvironment influences disease progression will yield crucial understandings in how to target melanoma. The impacts of understanding these interactions are clinically important with recent advances in immunotherapy. The zebrafish has innate and adaptive immune systems, the latter replete with B-cells, T-cells, regulatory T-cells, and other cell subpopulations [107,108,109,110]. In addition, zebrafish melanomas form, much like early-stage human tumors, in apposition to keratinocytes, fibroblasts, resident immune cells, vasculature, and adipocytes. This juxtaposition of cell types and ability to perform intravital imaging open many opportunities for melanoma studies in zebrafish. Similarly, zebrafish can be used to assay broad macroenvironmental choices and their impact on oncogenesis. For instance, by monitoring *crestin:EGFP* patches in a *Tg(mitfa:BRAF^V600E^);p53(lf)* strain, Grigura and colleagues observed that feeding amount significantly alters tumor onset [111]. Further understanding of the extrinsic factors driving melanoma will be vital to understanding how to prevent and treat metastatic disease.

### 8.2. Understanding the Role of Developmental Mechanisms in Melanoma Pathogenesis

In addition to insights gained by studying the tumor microenvironment, studies of melanocyte development can inform mechanisms that may be involved in melanoma pathogenesis. Complementing the discovery that GDF6-activated BMP signaling suppresses differentiation to promote invasive melanoma, Gramann and colleagues found that loss of the zebrafish ortholog *gdf6a* in development leads to an excess of melanocytes specified from the neural crest [112]. Similarly, Lister and colleagues demonstrated that MITF levels, which they directly controlled with the temperature-sensitive hypomorphic *mitfa(vc7)* allele, are critical to melanocyte development and regeneration in addition to the role in melanoma described above [45]. Understanding the role of these genes in melanocyte development provides insight into their mechanisms of action and suitability as melanoma therapeutic targets.

### 8.3. Utilizing Zebrafish Xenografts to Model Patient Disease

Zebrafish xenografts have been, and continue to be, a critical tool in understanding melanomagenesis [88,89,113,114]. During embryogenesis, hundreds of cells can be engrafted in the absence of an adaptive immune system [115]. Their transparency and many available transgenic reporters make zebrafish embryos an excellent system for performing xenografts. Furthermore, the small size of zebrafish embryos enables high-throughput screens of potential therapeutics. However, this small size also hinders the use of zebrafish embryos in studying heterogenous bulk tumors or invasion of melanoma cells into mature organs. There is also a temperature incompatibility between zebrafish embryos, which grow at a maximum of 35 °C, and patient derived xenografts (PDXs), which grow at 37 °C.

Adult zebrafish hosts have been used to overcome limitations of embryo xenografts. Irradiated adults have been used as hosts, but the recovery of adaptive immunity approximately three weeks after irradiation leads to death of engrafted cells, preventing long-term engraftment [113,116]. More recently, genetic immune cell knockouts grown at 37 °C were successfully developed and used to test therapeutic efficacy [117,118,119]. These zebrafish allow for long-term engraftment of patient derived samples. Now, in a clinically relevant timeframe, a patient’s melanoma could be engrafted, expanded, and tested with various therapies, helping to anticipate drug resistance patterns and revealing the best course of treatment for the patient. These new zebrafish PDXs can serve as a suitable complement to conventional mouse PDXs, offering time savings and higher throughput. These recent developments provide a unique opportunity to provide a direct clinical correlate of therapy.

### 8.4. Utilizing Zebrafish as a Screening Platform

Zebrafish have been used extensively for large-scale and targeted drug screens [92,120,121,122,123]. Their high fecundity and simple water-borne drug administration provide an ideal platform for high-throughput screening of drug panels. Additionally, their optical features, plentiful tissue-specific reporters, and conserved developmental and disease programs make phenotypic readouts straightforward and findings applicable to other species. Combinations of these tools and features has and will facilitate the discovery of novel melanoma therapeutics from existing drug libraries. For instance, a recent screen for drugs that abrogated the development of excess melanocytes in *kita:HRAS* embryos identified clotrimazole as a potential novel melanoma therapeutic [121]. Extending these screens to a setting with tumors as a direct readout is a beneficial next step, one that would greatly facilitate the identification of therapeutic lead compounds.

## 9. Conclusions

While significant progress has been made in the treatment of melanoma, advanced melanoma is still a devasting disease with poor outcomes. Zebrafish offer an excellent system for modeling melanocyte and melanoma biology. Recent advancements in the multifactorial determinants of melanoma, coupled with advances in genome editing and other technologies, present even greater opportunities to model melanoma and develop future therapeutics in this model system.
